# In Vitro Evaluation of Rigosertib Antitumoral and Radiosensitizing Effects against Human Cholangiocarcinoma Cells

**DOI:** 10.3390/ijms22158230

**Published:** 2021-07-30

**Authors:** Alessio Malacrida, Roberta Rigolio, Luigi Celio, Silvia Damian, Guido Cavaletti, Vincenzo Mazzaferro, Mariarosaria Miloso

**Affiliations:** 1 Experimental Neurology Unit, School of Medicine and Surgery, University of Milano—Bicocca, Via Cadore 48, 20900 Monza, Italy; roberta.rigolio@unimib.it (R.R.); guido.cavaletti@unimib.it (G.C.); mariarosaria.miloso@unimib.it (M.M.); 2 Medical Oncology Unit, ASST del Garda, 25015 Desenzano del Garda, Italy; luigi.celio@asst-garda.it; 3 Medical Oncology Department, Fondazione IRCCS Istituto Nazionale dei Tumori—Via Giacomo Venezian 1, 20133 Milano, Italy; silvia.damian@istitutotumori.mi.it; 4 Department of Hepatology, Hepato Pancreatic Biliary Surgery and Liver Transplantation, Istituto Nazionale Tumori, Foundation IRCCS, 20133 Milan, Italy; vincenzo.mazzaferro@istitutotumori.mi.it; 5 Department of Oncology, University of Milan, 20122 Milan, Italy

**Keywords:** Rigosertib, cholangiocarcinoma, chemotherapy, radiotherapy, mitotic catastrophe, cell cycle, autophagy, proteasome, cell migration

## Abstract

Cholangiocarcinoma is the first most common cancer of the biliary tract. To date, surgical resection is the only potentially curative option, but it is possible only for a limited percentage of patients, and in any case survival rate is quite low. Moreover, cholangiocarcinoma is often chemotherapy-resistant, and the only drug with a significant benefit for patient’s survival is Gemcitabine. It is necessary to find new drugs or combination therapies to treat nonresectable cholangiocarcinoma and improve the overall survival rate of patients. In this work, we evaluate in vitro the antitumoral effects of Rigosertib, a multi-kinase inhibitor in clinical development, against cholangiocarcinoma EGI-1 cell lines. Rigosertib impairs EGI-1 cell viability in a dose- and time-dependent manner, reversibility is dose-dependent, and significant morphological and nuclear alterations occur. Moreover, Rigosertib induces the arrest of the cell cycle in the G2/M phase, increases autophagy, and inhibits proteasome, cell migration, and invasion. Lastly, Rigosertib shows to be a stronger radiosensitizer than Gemcitabine and 5-Fluorouracil. In conclusion, Rigosertib could be a potential therapeutic option, alone or in combination with radiations, for nonresectable patients with cholangiocarcinoma.

## 1. Introduction

Rigosertib (ON-01910) (Rig) is a non-ATP competitive multi-kinase inhibitor in clinical development. It is an antimitotic agent that induces G2/M cell cycle arrest [1]. Polo-like kinase 1 (PLK1) is the main target of Rig, and its inhibition could induce cell cycle arrest in the G2/M phase, mitotic failure and catastrophe, and cell death, by regulating G2/M checkpoints proteins, spindle assembly, and centrosome maturation [1,2,3]. Alterations induced by Rig offer the ideal conditions for radiotherapy treatment, because, in the G2/M phase, cells are more sensitive to radiation [4]. In recent work, Agoni et al. demonstrated that the combination of Rig with radiation therapy increased the efficacy of the treatment on HeLa cervical cancer cells, and it was a more effective radiosensitizer than cisplatin in vitro [5]. In the present work, we evaluated the effects of Rig on a cholangiocarcinoma cell line, EGI-1 alone or in combination with radiations. Moreover, we have evaluated Rig effects also in other cholangiocarcinoma cell lines, human TFK-1, and results are presented in Supplementary Materials.

Cholangiocarcinoma (CCA) is the first most common cancer of the biliary tract and the second most common hepatic malignancy. Although surgical resection remains the only potentially curative treatment option for CCA patients, radical surgery is possible for only a small proportion of cases. Moreover, it has been observed that up to 50% of patients deemed resectable at diagnosis are found to be unresectable during exploratory laparotomy. Anyway, surgical resection has a five-year survival rate of 30% [6]. Nonresectable CCA has few therapeutic options, and it is often considered chemotherapy-resistant. One of the most used and effective drug available is Gemcitabine (Gem), and in the last years, a phase III clinical trial demonstrated that a combination of GEM and cisplatin increased the survival rate of patients [6]. Other locoregional therapies, including chemo-embolization and radio-embolization, or radiotherapy in combination with Gem or 5-Fluorouracil (5-FU), could potentially increase patient survival, but to date, they have not yet demonstrated significant survival benefits [7].

A crucial problem for CCA is that his diagnosis occurs too late when metastases have already reached other organs. This is mainly caused by a strong and early ability of CCA to metastasize. This limits the ability to surgically treat the tumor. Already in the early stages of the disease, liver and lymph nodes may present CCA metastases. As the tumor progresses, other organs, such as the lungs, bones, and brain, may be affected by metastasis [8,9]. Cholangiocarcinoma is a rare tumor, considered an orphan tumor (retrieved July 2021, from https://www.orpha.net). Therefore, it becomes essential to find new drugs or combination therapies to treat nonresectable CCA and improve the overall survival rate of patients. 

In this article, we have analyzed in vitro the antitumoral effects of Rig on the EGI-1 CCA cell line either as a single agent or in combination with radiation treatment, to demonstrate if the treatment could potentially be more effective than those currently available with Gem and 5-FU.

## 2. Results

### 2.1. Evaluation of Rigosertib, Gemcitabine and 5-Fluorouracil Effects on Cell Viability of EGI-1 Cholangiocarcinoma Cells

**Figure 1:**
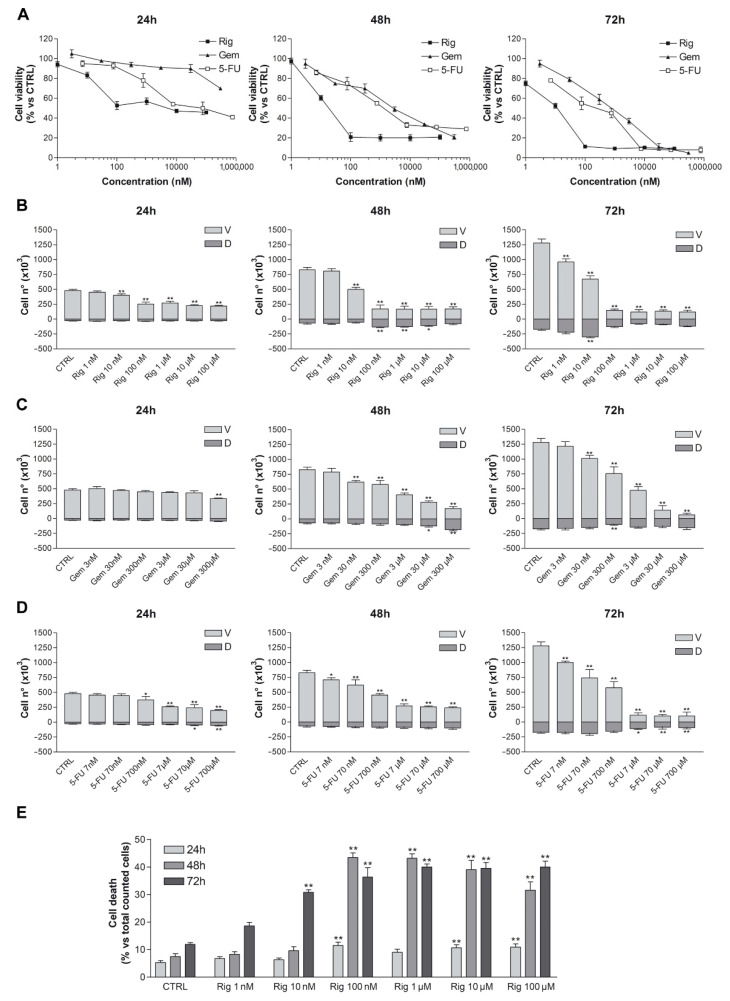
Trypan blue vital count of EGI-1 cells treated with Rig, Gem, or 5-FU. (**A**) Percentage of viable cells after treatment with different concentrations of Rig (1 nM–100 µM), Gem (3 nM–300 µM), and 5-FU (7 nM–700 µM). (**B**) Number of viable (V) and dead (D) cells treated with increasing concentrations of Rig (1 nM–100 µM) for 24, 48, and 72 h. (**C**) Number of viable (V) and dead (D) cells treated with increasing concentrations of Gem (3 nM–300 µM) for 24, 48, and 72 h. (**D**) Number of viable (V) and dead (D) cells treated with increasing concentrations of 5-FU (7 nM–700 µM) for 24, 48, and 72 h. (**E**) Percentage of counted EGI-1 death cells after Rig treatment. The percentage is calculated on the total number of counted cells. Data are presented as the mean ± SD of at least three independent experiments (* *p* < 0.05, ** *p* < 0.01 vs. CTRL).

Trypan blue vital count was used to evaluate the effect of Rig, Gem, and 5-FU on EGI-1 cell viability and cell death. EGI-1 cells were treated with increasing concentrations of Rig (1 nM–100 µM), Gem (3 nM–300 µM), and 5-FU (7 nM–700 µM). After 24, 48, and 72 h, cells were harvested and stained with trypan blue dye. Rig had a dose- and time-dependent effect on EGI-1 cell viability when compared to untreated EGI-1 cells (CTRL), reaching a maximum effect at 100 nM. At higher concentrations, results were comparable to Rig 100 nM. Rig was more effective than both Gem and 5-FU at all evaluated times. Its IC_50_, after 24 h of treatment, was 160 ± 15 nM, while Gem and 5-FU IC_50_ were >300 µM and 76 ± 10 µM, respectively (Figure 1). 

At 24 h the percentage of dead cells after Rig 1 and 100 nM treatment was almost comparable to the one obtained with untreated control cells (5% vs. 11%), while for Rig 100 nM, 1, 10 and 100 µM is observed a significant increase. At 48 h, the percentage of dead cells after Rig 100 nM and 1 µM is very similar (about 45%), while in Rig 10 and 100 µM, the percentage reduced, probably due to an intrinsic reduction in the number of live cells. However, in all these concentrations, the obtained percentage of cell death is always significantly increased compared to CTRL cells. At 72 h, a higher percentage of cell death is observed after Rig 1–10 and 100 µM treatment. For Rig 10 and 100 nm, the percentage of cell death is significantly increased compared to CTRL, but is lower than other concentrations. Moreover, the percentage of dead cells after 72 h of treatment with Gem 300 µM and 5-FU 700 µM represented 70% and 48%, respectively (Supplementary Table S1).

### 2.2. Rigosertib Reversibility

**Figure 2:**
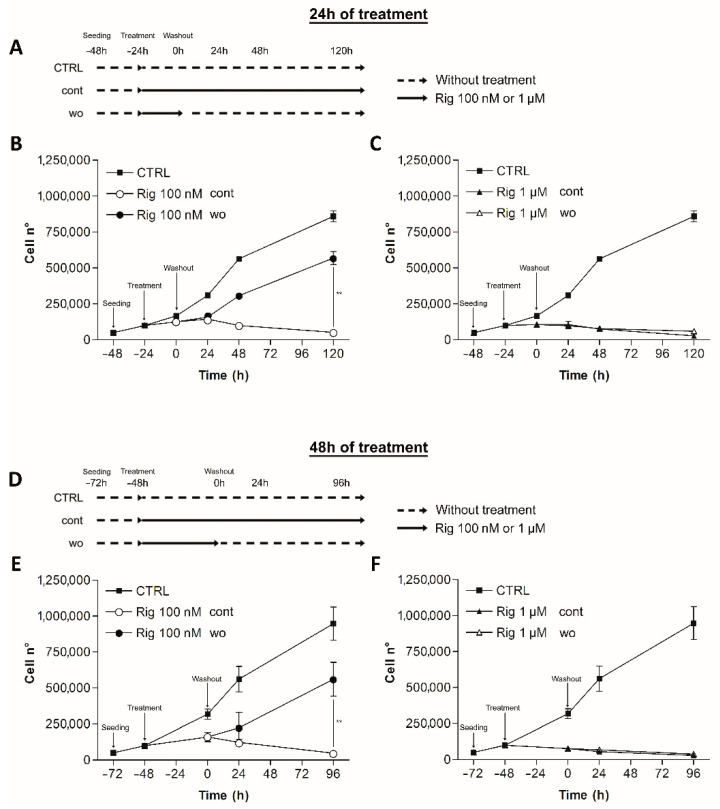
Reversibility of Rig against cell viability of EGI-1 cells. (**A**–**D**) Time schedules of Rig treatments. Black arrows represent the presence of Rig (100 nM or 1 µM) in the culture medium of continuous (cont) or washout (wo) treatments. Dashed lines represent culture medium without treatment (CTRL). (**B**,**C**) EGI-1 cells were treated with Rig 100 nM (**B**) or 1 µM (**C**). After 24 h of Rig treatment, the medium was changed with fresh medium with or without Rig (cont or wo) (**E**,**F**) EGI-1 cells were treated with Rig 100 nM (**E**) or 1 µM (**F**). After 48 h of Rig treatment, the medium was changed with fresh medium with or without Rig (cont or wo). Both after 24 or 48 h of Rig treatment the number of viable cells were counted using trypan blue 24, 48, 120 h after the medium change. EG-1 cells continuously treated with Rig (cont) and EG-1 cells without any type of treatment (CTRL) represent, respectively, positive and negative controls. Data are represented as the mean ± SD of at least three independent experiments (** *p* < 0.01 Rig cont vs. Rig wo).

Rig reversibility was assessed by trypan blue vital count. EGI-1 cells were treated with Rig 100 nM or 1 µM, concentrations that achieve the maximum effect of Rig against EGI-1 cells. After 24 and 48 h of treatment, the culture medium was replaced by fresh medium without Rig (wo). EGI-1 cells with Rig continuous treatment (cont) or without any type of treatment (CTRL) were used as controls. Cells were then counted at different time points (Figure 2A,D).

Rig 100 nM effect on cell viability was reversible. Cells treated with Rig for 24 or 48 h, restarted to grow after the medium change. The number of cells was higher than cells treated with Rig continuously, but was lower than untreated controls. 

Rig 1 µM effect instead was not reversible at considered time points. Cells could not restart growing, and the growth curve was comparable to the one obtained without removing Rig from the culture medium (Figure 2).

### 2.3. Rigosertib Alters EG-1 Cell Morphology, Cell Size and Nuclei Number

**Figure 3:**
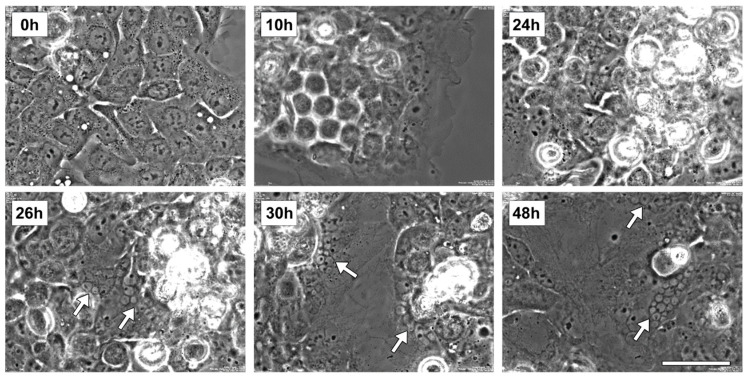
Live imaging of EGI-1 cells treated with Rig. Images representative of EGI-1 cells treated with Rig 100 nM for 48 h. Images represent the most important events observed during the study and are representative of three independent experiments. White arrows indicate micronucleated cells. Scale bar = 40 µm.

Preliminar live imaging studies were performed to evaluate the morphological alteration of EGI-1 cells following Rig 100 nM treatment (the concentration able to a maximum effect on cell viability). The representative images of Figure 3 show the most important events observed during the experiment. At start time (0 h), EGI-1 cells had a normal epithelioid shape and were mononucleated. After 10 h of Rig treatment cells became 3D-round-shaped (indicative of attempted mitosis), and this alteration persisted until 24 h of treatment. At 26 h, the first micronucleated cells appeared, and round-shaped cells were reattached to the dish surface. At 30 h, cells expanded their size, and increased the number of micronucleated cells was evident. These aspects also persisted at 48 h.

Based on these results, we have performed morphology experiments treating EGI-1 cells with different concentrations of Rig (10 nM, 100 nM, and 1 µM) for 24, 48, and 72 h. Cells size was measured, and the number of nuclei was counted. 

**Figure 4:**
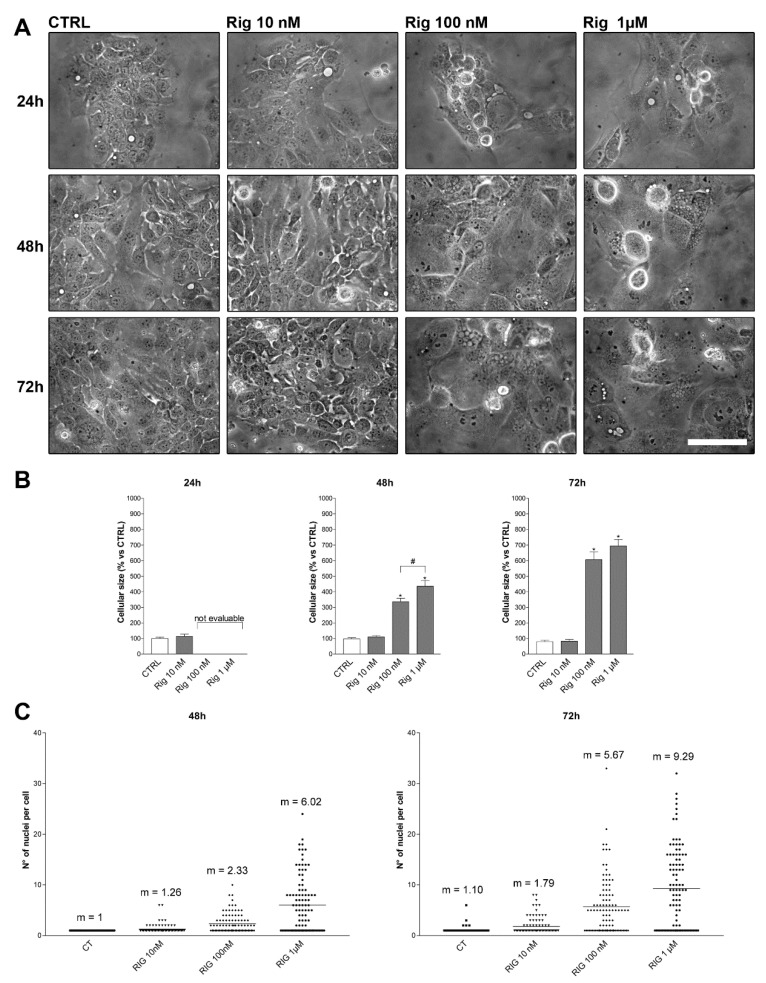
Cell size and nuclei number of EGI-1 cells treated with Rig. (**A**) Representative images of EGI-1 cells untreated or treated with different concentrations of Rig (10 nM–100 nM–1 µM) for 24, 48, and 72 h. Scale bar 40 µm. (**B**) Graphs represent the mean ± SD size of cells treated with increasing concentrations of Rig (1 nM–100 µM for 24, 48, and 72 h. (**C**) Graphs represent the number of nuclei/micronuclei counted in each EGI-1 cell (at least 100 cells counted for each condition) treated with increasing concentration of Rig (10 nM–1 µM). The horizontal black line and the number on the top represent the mean number of nuclei/micronuclei. (* *p* < 0.01 vs. CTRL; # *p* < 0.05 vs. Rig 100 nM).

After 24 h of treatment, cell size and nuclei number were not evaluable, due to round-cell-shape for Rig concentrations higher than 10 nM. As observed in live imaging experiments, after 26–30 h of treatment, cells were reattached to the dish surface and returned measurable. After 48 h and 72 h of Rig treatment, EGI-1 cells enlarged their size in a dose- and time-dependent manner when compared to untreated control cells (Figure 4A,B). 

Untreated control cells were mono-nucleated, and after 72 h, only a few cells were bi-nucleated. Rig treatment induced a dose- and time-dependent increase of micronuclei number. EGI-1 cells treated with Rig 100 nM at 48 and 72 h had an average number of micronuclei of 2.33 and 5.67 for Rig 100 nM at 48 and 72 h, respectively. EG-1 cells treated with Rig 1 µM at 48 and 72 h presented an average number of micronuclei was of 6.02 and 9.29 at 48 and 72 h, respectively (Figure 4A,C).

### 2.4. GIEMSA Staining of EGI-1 Cells Treated with Rigosertib

**Figure 5:**
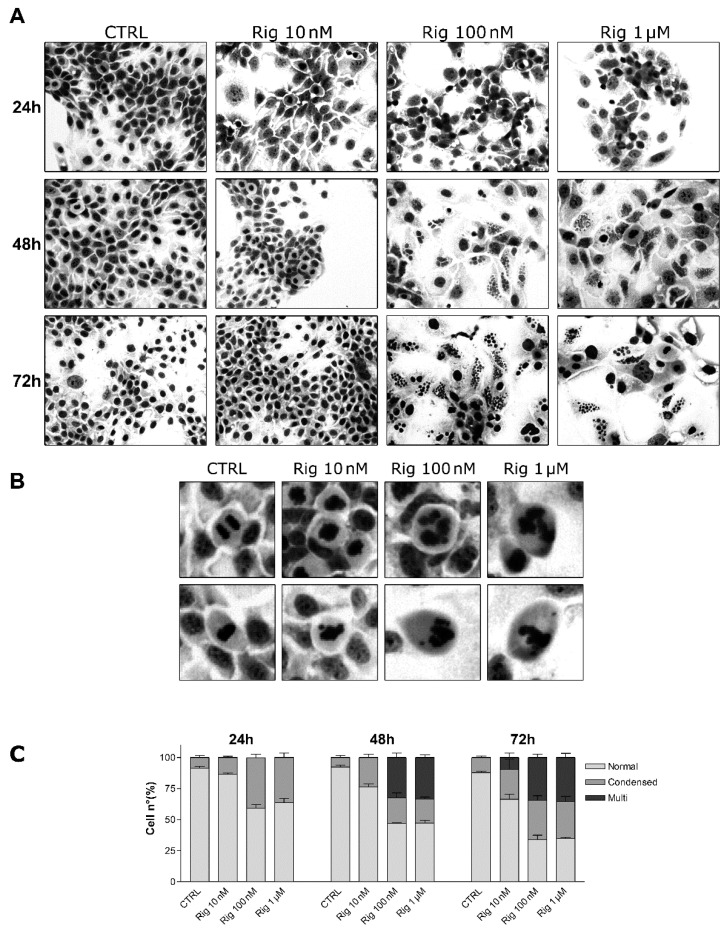
GIEMSA staining of EGI-1 cells treated with Rig. (**A**) Representative images of EGI-1 cells untreated or treated with Rig (10 nM, 100 nM, 1 µM) for 24, 48, and 72 h. (**B**) Representative magnified images of EGI-1 cells untreated or treated with Rig (10 nM, 100 nM, 1 µM) for 24 h. (**C**) The graph represents the mean ± SD percentage of cells with normal nuclei (normal), condensed nuclei (condensed), or micronucleated (micro), after treatment with increasing concentrations of Rig (10 nM–1 µM) for 24, 48, and 72 h. Data are presented as the mean ± SD of three independent experiments.

Nuclei morphology and micronucleation were evaluated by GIEMSA staining of EGI-1 cells. Cells were treated with different concentrations of Rig (10 nM, 100 nM, and 1 µM). After 24, 48, and 72 h, cells were stained with GIEMSA dye. At Rig 10 nM, condensed chromosomes and micronuclei were observed, respectively, only after 48 and 72 h of treatment. Differently, in the presence of Rig 100 nM and 1 µM, condensed chromosomes were evident after 24 h of treatment. Instead, micronucleated cells became visible after 48 h of Rig (Figure 5). Comparing 48 and 72 h of treatment, both for Rig 100 nM and 1 µM, the percentage of EGI-1 cells with normal nuclei decreased, while those with condensed nuclei increased. The percentage of cells with micronuclei remained constant.

Moreover, untreated mitotic cells had normal condensed nuclei in prometaphase, and the segregation of sister chromatids occurred in an orderly manner in anaphase. Cells treated with Rig 10 nM slightly lost the ordered organization seen in control cells, but the alterations were not significant. On the contrary, cells treated with Rig 100 nM and 1 µM showed very evident alterations of condensed nuclei and in the segregation of the sister chromatids, which were very disordered and disorganized (Figure 5B).

### 2.5. Cell Cycle Analysis of EGI-1 Cells Treated with Rigosertib 

**Figure 6:**
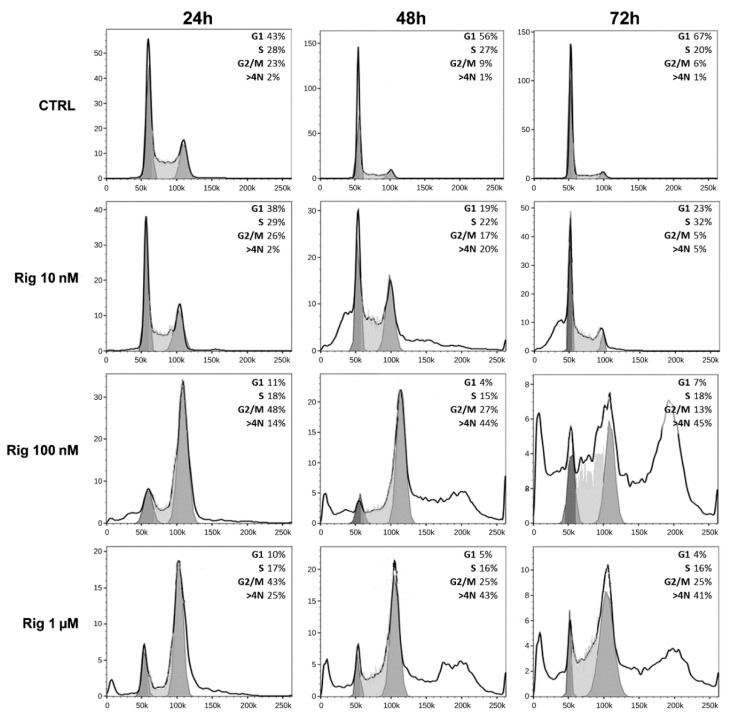
Cell cycle analysis of EGI-1 cells treated with Rig. Representative histograms of the distribution of EGI-1 cells in the different phases of the cell cycle after treatment with Rig 10 nM, 100 nM, and 1 µM, for 24, 48, and 72 h.

To analyze cell cycle modifications, EGI-1 cells were treated with Rig (10 nM, 100 nM, and 1 µM), and after 24, 48, and 72 h, cells were collected and analyzed by FACS (Figure 6). 

Untreated EGI-1 control cells (CTRL) at 24 h had a normal distribution in the cell cycle. At later times (48 and 72 h), cells started to stop in the G1 phase, a sign of confluence. However, at all times evaluated, control cells had only a minimal percentage of polyploid cells (less than 2%). 

Rig treatment induced a dose-dependent alteration in the cell cycle distribution. At 24 h, Rig 10 nM was comparable to CTRL, while at 48 h there was a significant increase in the percentage of polyploid cells (about 20%). At 72 h, both G2/M and polyploid cells were reduced, and a slight increase in G1/M was observed. Rig 100 nM and 1 µM had a comparable effect on the cell cycle. At 24 h, they induced a block in the G2/M phase and increased the number of polyploid cells compared to CTRL. At 48 and 72 h, the percentage of cells blocked in G2/M was reduced, but the number of polyploid cells significantly increased.

### 2.6. Rigosertib Induces Autophagy and Impairs Proteasome Activity of EGI-1 Cells

To evaluate the effect of Rig on autophagy and proteasome activity, EGI-1 cells were treated with Rig 10 nM, 100 nM, and 1 µM for 24, 48, and 72 h.

**Figure 7:**
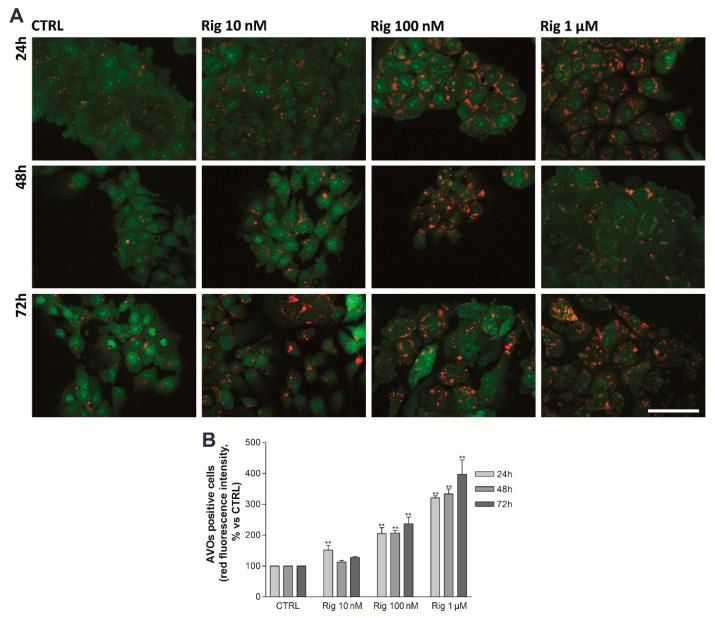
EGI-1 cells autophagy activity after Rig treatment. (**A**) Representative images of EGI-1 cells untreated (CTRL) or treated with different concentrations of Rig (10 nM, 100 nM, and 1 µM) at different times (24-48-72 h). Cells were stained with AO 1 µg/mL, and images were taken under a fluorescent microscope. Cytosol and nuclei are represented in green, while red represents AVOs. Scale bar 50 µm. (**B**) The graph represents the percentage of AVOs positive cells described in (**A**). Data are represented as the mean red fluorescence ± SD of three independent experiments. The red fluorescence intensity of CTRL is arbitrarily set to 100%. (** *p* < 0.01 vs. CTRL).

An autophagy process in EGI-1 cells was analyzed by Acridine Orange staining. Untreated control cells have a basal autophagy activity, and acidic vesicular organelles (AVOs) are observable in almost all cells. Rig treatment induces the enhancement of autophagy in a dose-dependent manner, while no significant increase was observed over time (Figure 7). 

**Figure 8:**
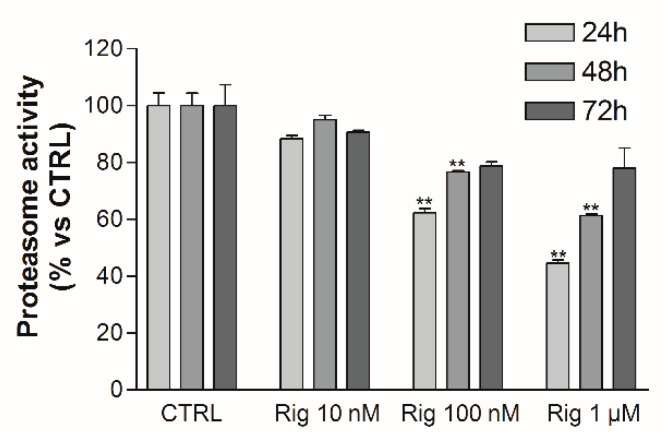
EGI-1 cells proteasome activity after Rig treatment. Proteasome activity of EGI-1 cells untreated (CTRL) or treated with different concentrations of Rig (10 nM, 100 nM, and 1 µM) at different times (24-48-72 h). The graph represents the mean ± SD of proteasome activity normalized to untreated controls set at 100%. (** *p* < 0.01 vs. CTRL).

Proteasome activity was evaluated by a fluorescent assay. Rig reduced proteasome activity in a dose-dependent manner after 24 h of treatment. At 48 h and 72 h, the proteasome was still inhibited in a dose-dependent manner by Rig, but the inhibition was lower than the one observed after 24 h of treatment (Figure 8). 

### 2.7. Rigosertib Impairs Cell Motility and Migration/Invasion of EGI-1 Cells

**Figure 9:**
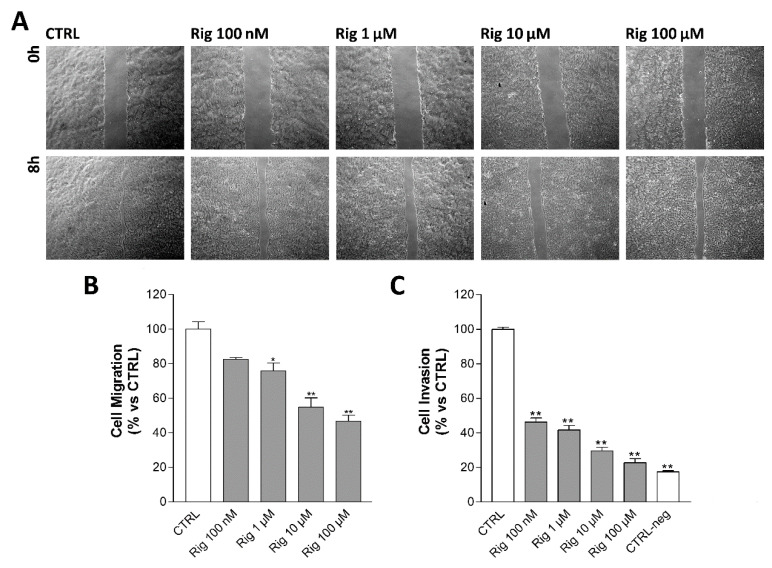
EGI-1 cell migration and invasion. (**A**) Representative images and (**B**) respective graph of the scratch wound healing assay of EGI-1 cells treated with increasing concentrations of Rig. The graph represents the mean ± SD percentage of the area of cells that were able to close the scratch after treatment with increasing concentration of Rig compared to corresponding untreated control cells. (**C**) Boyden chamber assay of EGI-1 cells treated with increasing concentrations of Rig. The graph represents the percentage of cells that can pass through the membrane. CTRL and CTRL-neg represent cells without any treatment that passed through the membrane, respectively, in the presence or absence of serum in the low chamber. Graphs are the mean ± SD of three independent experiments. (* *p* < 0.05, ** *p* < 0.01 vs. CTRL).

Cell motility of EGI-1 cells treated with Rig was evaluated by Scratch wound healing assay. Cells were treated with Rig 10 nM, 100 nM, and 1 µM. After 24 h from the beginning of the assay, untreated control cells have almost completely closed the scratch. Rig impairs cell motility in a dose-dependent manner and slows down the closure of the scratch (Figure 9A). 

Cell invasion of EGI-1 cells treated with Rig was evaluated by Boyden chamber assay. Untreated control cells pass through the gelatin-coated membrane attracted by serum, while in negative controls, without serum as chemoattractant, only a few cells can pass. Despite the presence of serum, Rig reduced the number of cells that passed through the membrane in a dose-dependent manner (Figure 9B).

### 2.8. Evaluation of the Effects of Rigosertib and Radiations Combination on EGI-1 Cells 

**Figure 10:**
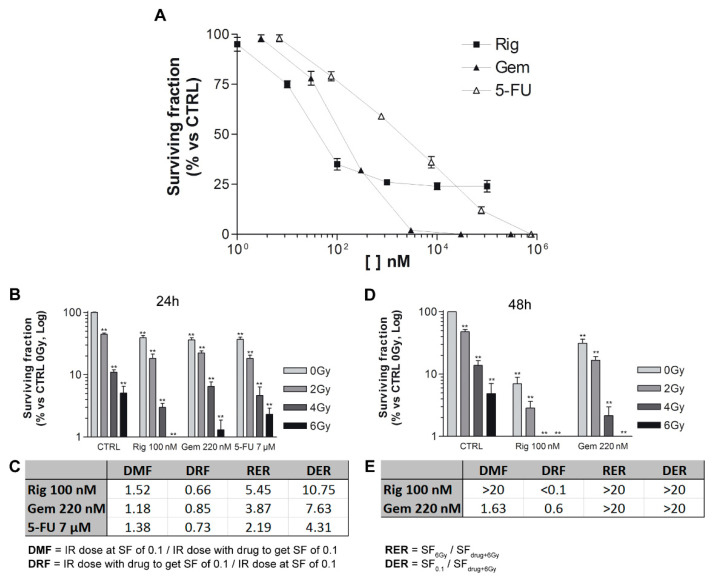
Clonogenic assay of EGI-1 cells and Rig. (**A**) Clonogenic assay of EGI-1 cells treated with increasing concentrations of Rig (1 nM–100 µM), Gem (3 nM–300 µM), and 5-FU (7 nM–700 µM) without radiations. (**B**) Clonogenic assay of EGI-1 cells treated with Rig 100 nM, Gem 220 nM, and 5-FU 7 µM for 24 h, and irradiated with increasing doses (0–6 Gy). (**C**) Results table of clonogenic assay of (**B**). DMF = Dose Modifying Factor, DRF = Dose Reduction Factor, RER = Radiation Enhancement Ratios and DER = Drug Enhancement Ratios, IR = irradiation, SF = surviving fraction. (**D**) Clonogenic assay of EGI-1 cells treated with Rig 100 nM and Gem 220 nM for 48 h, and irradiated with increasing doses (0–6 Gy). (**E**) Results table of clonogenic assay of (**D**). Graphs represent the mean ± SD surviving fraction of three independent experiments. (** *p* < 0.01 vs. CTRL).

To identify the concentrations of Rig, Gem, and 5-FU to use in radiation experiments, we have performed clonogenic assays without radiations. EGI-1 cells were treated for 24 h with increasing concentrations of Rig (1 nM–100 µM), Gem (3 nM–300 µM), and 5-FU (7 nM–700 µM). The clonogenic assay was performed immediately after the removal of drugs. After 12 days of incubation, untreated control cells were able to form colonies, while Rig reduced this ability in a dose-dependent manner, and a survival fraction of 30% (SF30) was obtained with Rig 100 nM. In addition, Gem and 5-FU reduced colony formation in a dose-dependent manner, and their SF30 are 220 nM and 7 µM, respectively. These concentrations were used in the following radiation experiments (Figure 10A). 

EGI-1 cells were treated with drugs for 24 h and then irradiated with different doses (0–6 Gy). Rig, Gem, and 5-FU, in combination with radiations, impaired the formation of EGI-1 colonies, and the effects were more significant than ones obtained with radiation- or drug-only treatments (Figure 10B). To compare the differences between the radiosensitizing effect of the three drugs, Dose Modifying Factor (DMF), Dose Reduction Factor (DRF), Radiation Enhancement Ratios (RER), and Drug Enhancement Ratios (DER) were calculated. DMF and DRF values were comparable between the three drugs, but Gem resulted in the most radiosensitizing drug, even if slightly. Contrariwise, RER and DER demonstrated that Rig was a more potent radiosensitizer than Gem and 5-FU for EGI-1 cells only at 4 and 6 Gy radiation (Figure 10C). 

Subsequently, we compared Rig with Gem, the drug that resulted in the most effective after 24 h of treatment. (Figure 10D). When cells were treated for 48 h, Rig was a more effective radiosensitizer than Gem. DMF and DRF values obtained with Rig were significantly better than the ones obtained with Gem. DER and RER, due to the high cytotoxic effect of both drugs at 6 Gy, are not evaluable and comparable (Figure 10E).

### 2.9. Evaluation of the Effects of Rigosertib on TFK-1 Cells

To investigate if Rig is also effective in a different human CCA cell line, we performed experiments in TFK-1 cells, a cell line, which, unlike EGI-1, does not appear to carry the KRAS gene mutation. 

All the results are comparable to the ones obtained with EGI-1 cells. More in detail, Rig impaired cell viability of TFK-1 in a dose- and time-dependent manner, and it was more effective than bot Gem and 5-FU (IC_50_ at 24 h were, respectively, 125 nM, 30 µM, and 1.5 µM) (Supplementary Figure S1). Moreover, Rig induced in TFK-1 the same morphological and nuclear alterations observed in EGI-1 cells: Increased cell size and micronuclei formation (Supplementary Figures S2 and S3). Cell cycle was blocked in the G2/M phase starting from the 100 nM concentration, similar to that observed with EGI-1 cells (Supplementary Figure S4). The only significant difference observed in TFK-1 cells, was that Rig induced the formation of a greater quantity of polyploid cells at 1 µM concentration, compared to EGI-1 cells. Another difference between the two cell lines is that Rig is slightly less effective in reducing cell migration and invasion of TFK-1 cells than of EGI-1 cells (Supplementary Figure S5). Lastly, Rig has the same radiosensitizing effect observed in EGI-1 cells against TFK-1 cells, although a higher concentration had to be used to obtain SF30 (Supplementary Figure S6).

## 3. Discussion

Cholangiocarcinoma is a very aggressive tumor, and to date, there are only a few therapeutic options available that can improve the overall survival of patients. Unfortunately, these therapies are often ineffective, due to the high chemoresistance and heterogeneity of CCA [10]. It is, therefore, necessary to identify new drugs or combination therapies that will improve the efficacy against CCA. 

Rig is a multi-kinase inhibitor that could be considered an alternative therapeutic option for different solid tumors and hematologic malignancies. Its efficacy against myelodysplastic syndrome, head and neck cancer, hepatocellular carcinoma, retinoblastoma, and glioblastoma has already been demonstrated [11,12,13,14,15,16,17,18]. Moreover, on the website https://clinicaltrials.gov/ (July 2021) it is possible to highlight that Rigosertib is involved in various clinical trials. Contrariwise, Rig does not appear to be toxic in normal human cells, as proved by Agoni et al. in normal human BJ fibroblast cells and normal human Ectocervix cells [5].

In this work, we have evaluated in vitro the antitumoral effect of Rig in CCA EGI-1 cells (and TFK-1 cells, see Supplementary Data) both alone and in combination with radiation.

First, Rig can impair the viability of EGI-1 cells in a dose- and time-dependent manner, and its IC_50_ (in the nanomolar range) are lower than 5-FU and Gem, the golden standard for the treatment of CCA. 

Moreover, Rig induces important alteration of EGI-1 cell morphology. Untreated control cells have normal epithelial-like morphology and a single nucleus (only in rare cases, some cells are bi-nucleated). Instead, Rig treatment induces significant changes in cell morphology. In the first 24 h of treatment, most of the cells became round-shaped (sign of the beginning of the mitosis process). It is widely demonstrated that when cells enter mitosis, a series of remarkable structural changes are triggered (condensation of chromosomes, separation of centrosomes, and formation of the microtubule spindle are just some of the most evident examples) [19]. In particular, in vitro, adherent-growing cells reduce adhesion to the substrate and round up to assume a characteristic spherical shape, necessary to facilitate a successful cell division [20]. If mitosis occurs successfully, the mother cell gives rise to two daughter cells in which nuclei reform with the correct shape and DNA content. Otherwise, mitosis failure prevents cell division, and the nucleus does not reform properly due to chromosome missagregation [21]. Consequently, cells acquire a large number of micronuclei. Furthermore, in the following hours, cells increase their size and assume an irregular shape (micronucleated giant cells) [22]. The morphological alterations observed in EGI-1 cells may suggest that cells have undergone mitotic catastrophe, an oncosuppressive mechanism that follows a mitotic failure [16,17]. The process will lead cells to an irreversible antiproliferative fate: death or senescence [23]. Our results suggest the induction of aberrant mitosis and the increase of nuclear DNA content in EGI-1 cells treated with Rig. These effects after Rig treatment have also been demonstrated in hematopoietic cell lines [24]. Instead, our results demonstrated for the first time that Rig treatment can increase the cellular size and induce the formation of micronuclei (multinucleated giant cells). In both processes, Rig 1 µM is more effective compared to Rig 100 nM. Vakifahmetoglu et al. affirm that one of the most prominent morphological characteristics of mitotic catastrophe is the formation of giant cells with abnormal nuclei [25]. Mitotic catastrophe could represent a mechanism of action by which Rig acts on CCA cells. This undoubtedly represents a novelty as, until now, there are no data demonstrating mitotic catastrophe as a mechanism of cell death in CCA cells treated with other antitumoral agents. Further investigations will be necessary to confirm our hypothesis. 

We also demonstrated for the first time that Rig can inhibit proteasome activity. Reduction of proteasome activity leads to accumulation of polyubiquitinated proteins, ER stress, and cell death [26]. Moreover, the proteasome is also involved in chromatin relaxation after mitosis [27]. A long-term proteasome inhibition, combined with the alterations induced by PLK1 inhibition [28], could have an important effect on the nuclear organization, and it could contribute to micronuclei formation [27]. In addition, it seems that micronuclei lack active proteasome machinery, and this situation could further increase the incorrect nuclear reorganization [29].

The role of autophagy as molecular mechanism involved in antitumoral effects is controversial [30,31,32]. In this work, we demonstrated for the first time that Rig induces a significant dose-dependent increase in the autophagy process. The presence of micronuclei could trigger a process of nucleophagy, a selective form of autophagy to restore cellular homeostasis [33]. Rello-Varona and colleagues demonstrated that autophagy could be involved in micronuclei clearance [34]. They reported that micronuclei arising from alterations in the cell cycle and cytokinesis can be degraded by autophagy, but the percentage of micronuclei that is subjected to degradation is relatively small. Moreover, autophagy induction is considered a modulator of cell death [35]. The significant increase in autophagy that we observed after Rig treatment, with concomitant proteasome inhibition, could contribute to the fate of EGI-1 cells [30].

Reversibility experiments have shown that Rig 100 nM has a reversible action on EGI-1 cells, while the effect of Rig 1 µM is irreversible. This dose-dependent reversibility is particularly anomalous and very interesting, since the two concentrations have the same effects on cell viability. However, it should be emphasized that compared to Rig 100 nM, Rig 1 µM induces a higher increase of cell size, number of micronuclei, percentage of ploidy at 24 h, autophagy, and proteasome inhibition. These results suggest that the mechanisms activated by EGI-1 cell to overcome the effects observed after treatment with Rig 100 nM (highlighted by restart cellular growing after Rig removal) cannot compensate for the effects of more effective Rig 1 µM. Consequently, cells are unable to resume growing also after removal of Rig 1 µM and undergo cell death. Clearance of micronuclei and recovery from polyploidization probably play an important role in 1 µM Rig irreversibility.

CCA is an aggressive tumor, the liver and lymph nodes are the most common sites of metastasis [36]. Tumor infiltration and metastasis further decrease the efficacy of anticancer therapy. For this reason, it is important to develop a drug able to reduce not only tumoral cell viability, but is also effective against tissue infiltration and metastasis. The metastatic cascade can be separated into three main processes: Invasion, intravasation, and extravasation [37]. Rig inhibition of cell motility and invasion are dose-dependent, but while Rig 100 nM reduces only cell invasion, Rig 1 µM inhibits both cell motility and invasion. Therefore Rig 1 µM can inhibit the first step of the metastatic cascade. These data confirm the greater effectiveness of Rig 1 µM and suggest that Rig treatment of CCA EGI-1 cells is effective not only to induce cell death, but also to inhibit the diffusion of tumoral cells. 

Research of radiosensitizing agents is a possible alternative to classic pharmacological therapies and could overcome the pharmacoresistance of some types of tumors [38]. In particular, CCA is a high drug-resistant cancer, and conventional chemotherapy is often not sufficient alone. A combination of drugs, such as Rig, with radiotherapy, could improve the efficacy of the treatment, induce cell death, and overcome pharmacoresistance. Moreover, it is well known in the literature that cells in the G2/M phase are more sensitive to the effects of ionizing radiation [39,40]. Our experiments demonstrate that both Rig 100 nM and 1 µM block EGI-1 cells in the G2/M phase of the cell cycle, and this makes Rig an optimal candidate as a radiosensitizer. In our work, Rig 100 nM (Rig concentration with reversible effect) has proven to be a potent radiosensitizer in vitro after 24 h of treatment, with overall results comparable with Gem and 5-FU, two anticancer drugs with proven radiosensitizing efficacy. But after 48 h of treatment, Rig becomes significantly more effective when compared to Gem. This could be due to the state of EGI-1 cell DNA following the treatment with Rig. As we demonstrated by cell cycle analysis, Rig induces arrest of EGI-1 cells in the G2/M phase after 24 h of treatment, while in the following hours, it increases the percentage of polyploid cells. In this condition, cells are probably blocked in the G2/M phase, but they have a quantity of DNA higher than 4 N. This higher quantity of DNA increases the radiosensitivity of cells and the damage of DNA [41]. Consequently, the combination of the Rig with radiotherapy obtains a significant increase in the killing of cells, eliminating even the cells that would have been resistant to one of the two therapies. Not only that, the combination of two therapies could make it possible in the future to use lower concentrations of drugs or radiations, reducing the possible side effects, while maintaining an adequate efficiency.

## 4. Materials and Methods

### 4.1. Cell Cultures and Materials

EGI-1 and TFK-1 CCA cells (DSMZ-German Collection of Microorganisms and Cell Cultures GmbH) were cultured in RPMI 1640 medium supplemented with 10% fetal bovine serum (FBS), 1% L-glutamine, and 1% Penicillin and Streptomycin (Euroclone, Pero, Italy). Cells were incubated at 37 °C and 5% CO_2_ in a humidified incubator.

Rig, kindly provided by Onconova Therapeutics (Newtown, PA, USA), Gem and 5-FU, were dissolved in water and then diluted directly in the culture medium to working concentrations. 

### 4.2. Trypan Blue Vital Count Assay

Trypan blue vital count assay was performed to quantify the number of viable and dead cells. All cell lines were plated in 6 well plates at 25 × 10^4^ cell/well density. After 24, 48, and 72 h of Rig treatment, adherent, and floating cells were harvested after trypsinization. Cells were then stained with Trypan blue dye (Sigma Aldrich, Burlington, MA, USA), and counted under a light microscope (Nikon Eclipse TS100). 

### 4.3. Reversibility 

Trypan blue vital count assay was also performed to assess the reversibility of Rig effect on cell viability. Cells were plated and treated as described in a previous paragraph. After 24 or 48 h, the culture medium with Rig was removed and replaced with fresh culture medium without treatments. Then, 24, 48, and 96 h after the medium change, cells were harvested, stained with Trypan blue dye, and counted. Cells with continuous Rig treatment were used to assess the reversibility of cell viability. 

### 4.4. Scratch Wound Healing Assay

A scratch wound healing assay was performed to evaluate cells motility. Cells were cultured in 6 well plate since 90% confluence. Complete medium was then replaced with serum free medium. After 16 h, a scratch with a 200 µL plastic tip mas made on the cell monolayer. After 2 washes with PBS, cells were treated with Rig for 24 h. Micrographs of scratches were taken at 0 and 24 h. Scratch areas were manually measured using ImageJ software, and migration areas were calculated, subtracting areas at 24 h to areas at 0 h.

### 4.5. Boyden Chamber Assay

Boyden chamber assay was performed to evaluate cell invasion of cells. The chamber consists of two compartments. In the lower compartment, a culture medium with 10% serum was placed. In the upper compartment, 5 × 10^3^ cells in serum-free medium were placed. The two compartments were separated by a gelatin-coated polycarbonate membrane with 8 µm pores (NeuroProbe, Gaithersburg, MD, USA). Rig was added in the upper chamber medium. After 8 h of treatment, the membrane was removed, fixed in methanol, and cells on the lower side were stained with Diff Quick kit (NeuroProbe, Gaithersburg, MD, USA). Membranes were then mounted on glass, and micrographs were taken. Image J software was used to manually quantify the number of migrated cells. Untreated cells were used as a positive control, while untreated cells without serum chemo-attractant were used as negative controls. 

### 4.6. Cell Morphology, Nuclei Count and Time-Lapse Imaging

EGI-1 cells were plated in 6 well plates and were treated with increasing concentrations of Rig. After 24, 48, and 72 h, micrographs were taken under a microscope. 

Time-lapse imaging was performed to evaluate morphological changes of EGI-1 cells. Cells were plated in 35 mm dishes at 1 × 10^5^ cells/dish density. After Rig treatment, micrographs of cells were taken every 5 min for 72 h in a Nikon Biostation. 

### 4.7. Giemsa Assay

Cells were plated in chamber-slide systems at 5 × 10^3^ cells/well density and were treated with increasing concentrations of Rig. After 24, 48, and 72 h of treatment, cells were stained with Giemsa: Cells were washed with PBS, and then fixed with methanol and acetic acid solution (3:1 ratio); wells were washed with phosphate buffer 0.12 M, stained with 4% Giemsa solution (Sigma Aldrich, Burlington, MA, USA), and washed with water. Micrographs of cells were taken and manually analyzed using ImageJ software. 

### 4.8. Cell Cycle Analysis

Cells were plated in 6 well plates at 25 × 10^4^ cells/well density and were treated with different concentrations of Rig. 

After 24, 48, and 72 h of treatments, cells were harvested using Trypsin. Cells were washed with PBS and resuspended in glucose/EDTA solution. Cells were then fixed with ethanol and stained with a PBS solution containing propidium iodide 20 µg/mL, and RNase 25 µg/mL. Cells were analyzed by FACS cytofluorimeter.

### 4.9. Acridine Orange Staining

Acridine orange (AO) staining was performed to assess cell autophagy. Cells were cultured in 6 well plates at a density of 25 × 10^4^ cells/well. After 24, 48, and 72 h of Rig treatment, cells were stained with AO at a final concentration of 1 µg/mL (Sigma Aldrich, Burlington, MA, USA), and micrographs were taken under a fluorescent microscope. Image analysis of fluorescence of each cell was performed using Image J software: Red fluorescence integrated density was measured for each cell, and then the value was normalized to cell area and background fluorescence. At least 100 cells for each condition were analyzed.

### 4.10. Proteasome Activity Assay

Proteasome activity of EGI-1 cells was assessed using N-Succinyl-Leu-Leu-Val-Tyr-7-Amido-4-Methylcoumarin fluorescent dye (Sigma Aldrich, Burlington, MA, USA). Cells were plated in 6 wells plates at a density of 25 × 10^4^ cells/well. After 24, 48, and 72 h, the total protein extract was obtained from cells using lysis buffer (Hepes 5 mM pH7.5, NaCl 150 mM, Glycerol 10%, Triton X100 1%, MgCl2 1.5 mM, EGTA 5 mM). The protein content of the extracts was quantified using Coomassie blue reagent (Euroclone, Pero, Italy). 

40 µg of proteins were added to a solution of 1x proteasome buffer and N-Succinyl-Leu-Leu-Val-Tyr-7-Amido-4-Methylcoumarin. After 2 h at 37 °C, fluorescence was measured in a microplate reader (Ex: 380 nm; Em: 460 nm).

### 4.11. Clonogenic Survival Assay and Irradiation

5 × 10^5^ EGI-1 cells were plated in T25 flask and treated with different concentrations of Rig, Gem, and 5-FU for 24 h. First, a clonogenic assay without radiation was performed to choose the concentrations of the three drugs with a survival rate of 30%. These concentrations were then used to perform the clonogenic assay in the presence of radiations. Cells were treated for 24 and 48 h and then exposed to different radiation doses (0-2-4-6 Gy).

For both experiments, without and with radiations, cells were harvested after trypsinization, counted, and plated in 100 mm dishes at low density to allow colony formation. After 10–12 days colony were fixed with methanol and stained with Giemsa solution. Photographs of whole dishes were taken, and colonies were counted using ImageJ software. 

To compare the differences between the radiosensitizing effect of the drugs, Dose Modifying Factor (DMF), Dose Reduction Factor (DRF), Radiation Enhancement Ratios (RER), and Drug Enhancement Ratios (DER) were calculated. DMF value is defined as the ratio between the radiation dose without and the radiation dose with the tested agent to achieve the same level of effect. DRF is defined as the ratio between the radiation dose with and the radiation dose without the tested agent to achieve the same level of effect. RER is defined as the ratio of surviving fractions obtained at 6 Gy alone and drug at 6 Gy. DER is defined as the ratio of surviving fractions at drug concentration, giving 10% survival and drug at 6 Gy.

### 4.12. Statistical Analysis

Data are reported as mean ± standard deviation (SD) from at least three independent experiments. Statistical analysis was performed using GraphPad Prism 3 software. The differences between control and treated cells were evaluated using One Way ANOVA analysis of variance followed by Dunnet’s multiple comparison test. Statistical significance was set at *p* < 0.05 or *p* < 0.01.

## 5. Conclusions

In conclusion, our work demonstrates that Rig in vitro is an effective antitumoral agent against the human CCA cell line EGI-1. Rig not only significantly reduced EGI-1 cell viability, but it can induce alteration in cell morphology and DNA content that, in combination with the inhibition of proteasome and increase of autophagy, leads to cell death. Moreover, Rig impairs cell motility and cell invasion, suggesting that Rig could be effective not only for treating cancer cells, but also for limiting their spread. 

Rig acts both as an antitumoral agent alone and as an effective radiosensitizer in combination whit radiation. 

Taken together, our data suggest that the Rig could be considered a very interesting potential therapeutic option for CCA treatment.

Further studies are essential to investigate the molecular pathways involved in its mechanisms of action. Moreover, in vivo studies will be needed to establish the safety and efficacy of Rig before clinical application in patients with CCA.

## Data Availability

Not applicable.

## Supplementary Materials

The following are available online at https://www.mdpi.com/article/10.3390/ijms22158230/s1.
