# Supplementary material for: In Vitro Evaluation of Rigosertib Antitumoral and Radiosensitizing Effects against Human Cholangiocarcinoma Cells
Source: Int J Mol Sci. 2021 Jul 30;22(15):8230. doi: 10.3390/ijms22158230 (PMC8348961; doi:10.3390/ijms22158230)
Supplement: Supplementary file 1 [file ijms-22-08230-s001.zip › ijms-1229033-supplementary.pdf]

In vitro evaluation of Rigosertib antitumoral and radiosensitizing effects against human cholangiocarcinoma cells

Alessio Malacrida, Roberta Rigolio, Luigi Celio, Silvia Damian, Guido Cavaletti, Vincenzo Mazzaferro, and Mariarosaria Miloso

Supplementary Table S1. Percentage of counted EGI-1 death cells after Gem and 5-FU treatments in Trypan blue assay (* *p* <0.05, ** *p* <0.01 vs CTRL).

**[Gem]**	**24h**	**48h**	**72h**
CTRL	5.3 ± 1.2	7.5 ± 1.8	11.9 ± 1.2
3nM	5.4 ± 1.1	8.2 ± 1.7	11.9 ± 1.8
30nM	5.0 ± 1.0	10.8 ± 3.0	12.9 ± 1.8
300nM	5.8 ± 1.2	12.8 ± 3.3	11.7 ± 1.7
3μM	6.4 ± 1.0	19.5 ± 2.4 **	23.4 ± 2.4 **
30μM	7.1 ± 1.1	29.9 ± 4.5 **	48.0 ± 9.2 **
300μM	11.8 ± 2.1 **	50.9 ± 5.9 **	70.8 ± 13.2 **

**[5-FU]**	**24h**	**48h**	**72h**
7nM	6.2 ± 1.2	9.5 ± 1.5	14.9 ± 2.1
70nM	7.8 ± 1.0	11.5 ± 2.1	20.8 ± 3.1
700nM	9.8 ± 2.6	15.7 ± 3.3	21.2 ± 2.9
7μM	12.5 ± 2.7 *	24.2 ± 5.8 **	48.8 ± 6.7 **
70μM	16.7 ± 3.1 **	27.0 ± 4.8 **	45.4 ± 18.7 **
700μM	21.8 ± 4.4 **	29.6 ± 6.2 **	48.12 ± 10.6 **

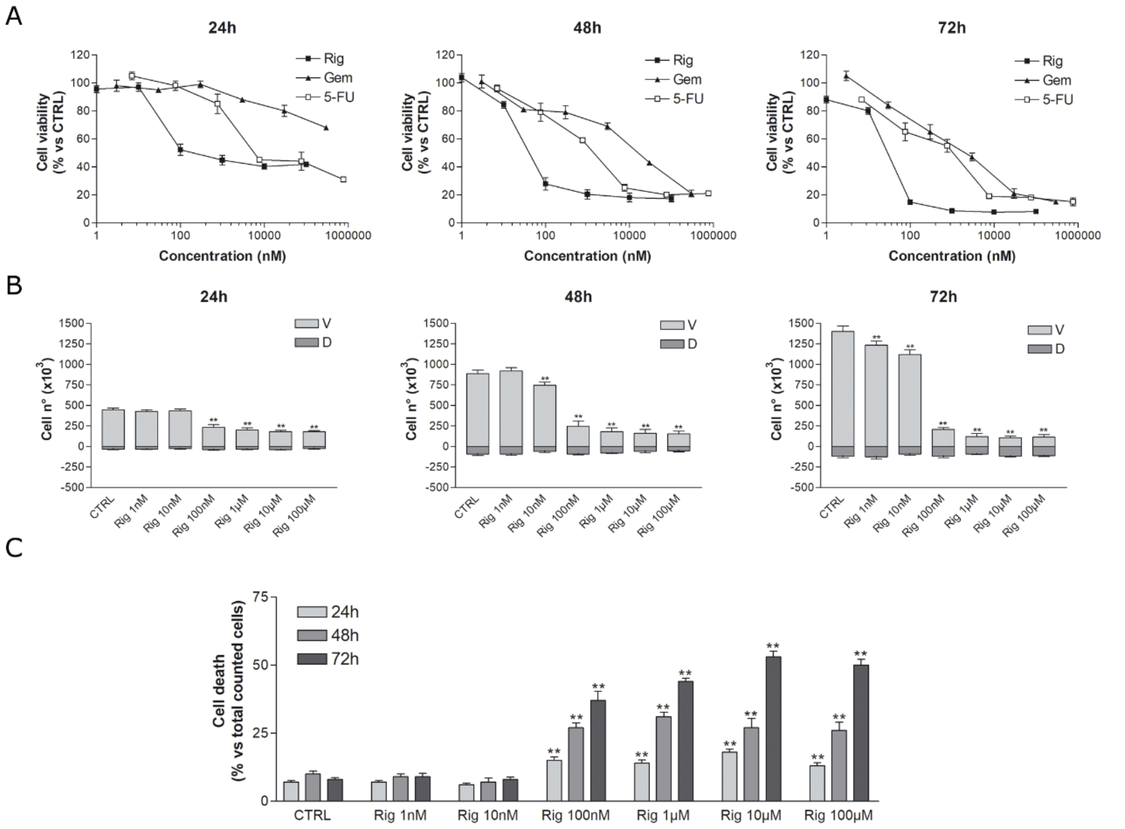

Supplementary Figure S1. . Trypan blue vital count of TFK-1 cells treated with Rig, Gem or 5-FU. (A) Percentage of viable cells after treatment with different concentrations of Rig (1nM – 100μM), Gem (3nM – 300μM) and 5-FU (7nM – 700μM). **(B)** Number of viable (V) and dead (D) cells treated with increasing concentrations of Rig (1nM – 100μM) for 24, 48 and 72h. **(C)** Percentage of counted TFK-1 death cells after Rig treatment. The percentage is calculated on the total number of counted cells. Data are presented as the mean ± SD of at least three independent experiments (** *p* <0.01 vs CTRL).

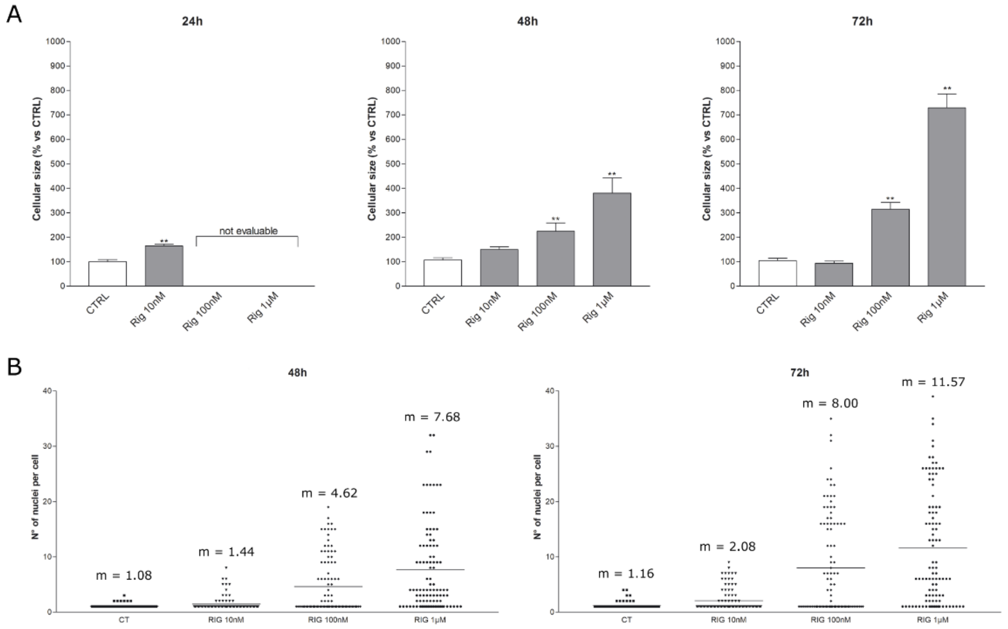

Supplementary Figure S2. Cell size and nuclei number of TFK-1 cells treated with Rig. (**A**) Graphs represent the mean ± SD size of cells treated with increasing concentrations of Rig (1nM – 100μM) for 24, 48 and 72h. (**B**) Graphs represent the number of nuclei/micro-nuclei counted in each TFK-1 cell (at least 100 cells counted for each condition) treated with increasing concentration of Rig (10nM – 1μM). Horizontal black line and the number on the top represents the mean number of nuclei/micro-nuclei. (p <0.01 vs CTRL).

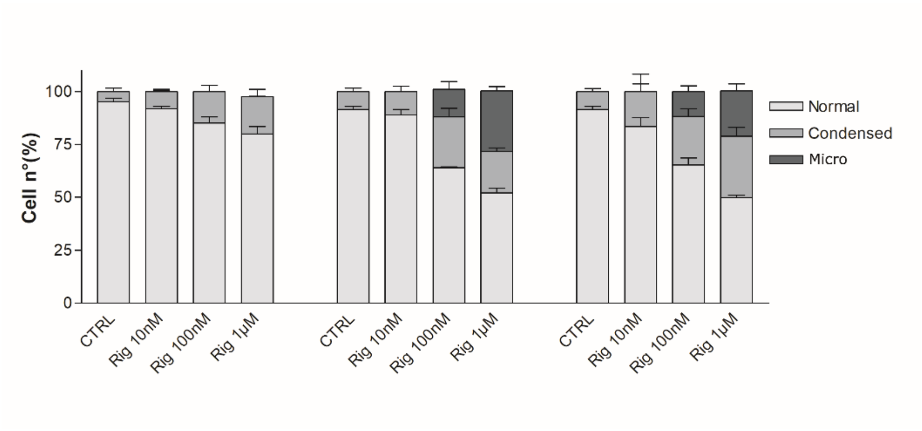

Supplementary Figure S3. GIEMSA staining of TFK-1 cells treated with Rig. Graph represent the mean ± SD percentage of cells with normal nuclei (normal), condensed nuclei (condensed) or micro-nucleated (micro), after treatment with increasing concentrations of Rig (10nM – 1μM) for 24, 48 and 72h. Data are presented as the mean ± SD of three independent experiments. (* p<0.05, ** p <0.01 vs CTRL).

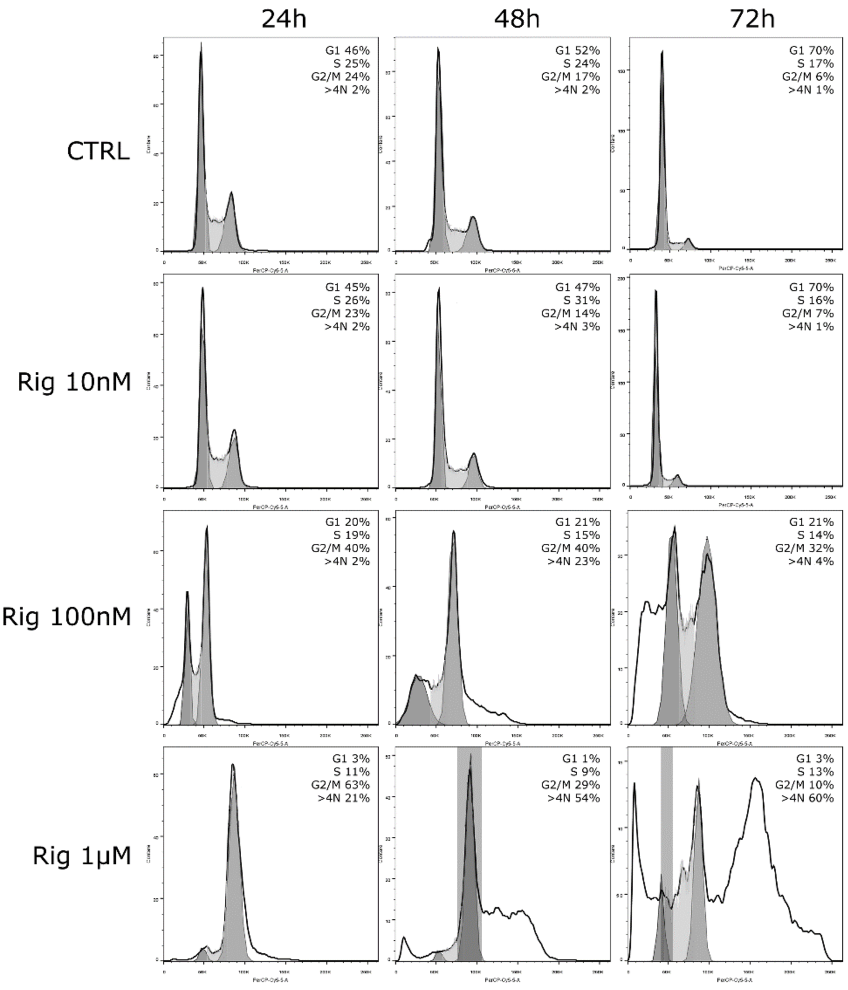

Supplementary Figure S4. Cell cycle analysis of TFK-1 cells treated with Rig. Representative histograms of the distribution of TFK-1 cells in the different phases of cell cycle after treatment with Rig 10nM, 100nM and 1µM, for 24, 48 and 72h.

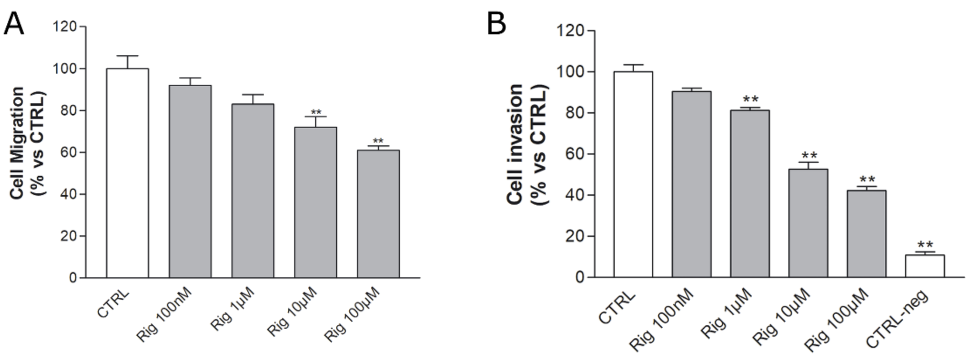

Supplementary Figure S5. TFK-1 cell migration and invasion. (A) Scratch wound healing assay of TFK-1 cells treated with increasing concentrations of Rig. The graph represent the mean ± SD percentage of the area of cells that were able to close the scratch after treatment with increasing concentration of Rig compared to corresponding untreated control cells. **(B)** Boyden chamber assay of TFK-1 cells treated with increasing concentrations of Rig. Graph represent the percentage of cells that are able to pass through the membrane. CTRL and CTRL-neg represents cells without any treatment that passed through the membrane respectively in presence or in absence of serum in low chamber. Graphs are the mean ± SD of three independent experiments. (* p <0.05, ** p <0.01 vs CTRL).

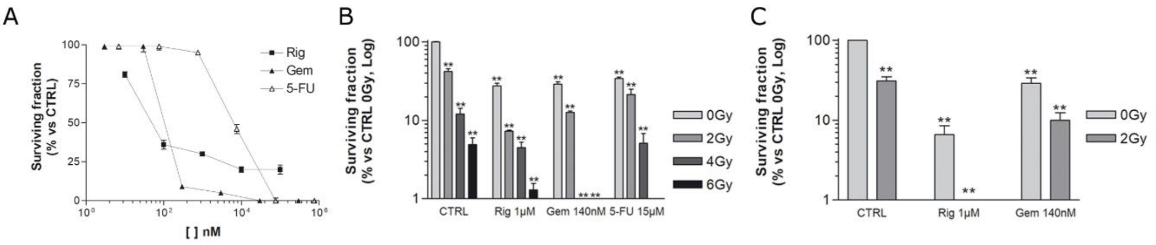

Supplementary Figure S6. Clonogenic assay of TFK-1 cells and Rig. (A) Clonogenic assay of TFK-1 cells treated with increasing concentrations of Rig (1nM-100μM), Gem (3nM-300μM) and 5-FU (7nM-700μM) without radiations. **(B)** Clonogenic assay of TFK-1 cells treated with Rig 1μM, Gem 140nM and 5-FU 15μM for 24h, and irradiated with increasing doses (0-6Gy). **(D)** Clonogenic assay of TFK-1 cells treated with Rig 1μM and Gem 140nM for 48h, and irradiated with increasing doses (0-6Gy). (** p<0.01 vs CTRL).
